# The March of the Beetles: Epistatic Components Dominate Divergence in Dispersal Tendency in *Tribolium castaneum*

**DOI:** 10.1093/jhered/esaa030

**Published:** 2020-08-14

**Authors:** Sarah N Ruckman, Heath Blackmon

**Affiliations:** 1 Department of Biology, Texas A&M University, 3258 TAMU, College Station, TX; 2 Ecology and Evolutionary Biology Interdisciplinary Program, Texas A&M University, 2475 TAMU, College Station, TX

**Keywords:** line cross-analysis, dispersal, epistasis, genetic components, life history

## Abstract

The genetic underpinnings of traits are rarely simple. Most traits of interest are instead the product of multiple genes acting in concert to determine the phenotype. This is particularly true for behavioral traits, like dispersal. Our investigation focuses on the genetic architecture of dispersal tendency in the red flour beetle, *Tribolium castaneum*. We used artificial selection to generate lines with either high or low dispersal tendency. Our populations responded quickly in the first generations of selection and almost all replicates had higher dispersal tendency in males than in females. These selection lines were used to create a total of 6 additional lines: F1 and reciprocal F1, as well as 4 types of backcrosses. We estimated the composite genetic effects that contribute to divergence in dispersal tendency among lines using line cross-analysis. We found variation in the dispersal tendency of our lines was best explained by autosomal additive and 3 epistatic components. Our results indicate that dispersal tendency is heritable, but much of the divergence in our selection lines was due to epistatic effects. These results are consistent with other life-history traits that are predicted to maintain more epistatic variance than additive variance and highlight the potential for epistatic variation to act as an adaptive reserve that may become visible to selection when a population is subdivided.

Complex traits are notoriously difficult to study, and dispersal behavior, which plays a significant role in life history, gene flow, and invasive species dynamics is no exception (Ronce 2007; Clobert et al. 2012; Travis et al. 2013). The genetics of dispersal tendency has been studied in a handful of birds and insects with narrow-sense heritability estimates encompassing the full range of heritability from 0 to 1 and an average of 0.35 (Clobert et al. 2012; Drangsholt et al. 2014; Saastamoinen et al. 2018). However, the number of genes associated with this behavior has only been studied in a few species with a range of results (Saastamoinen et al. 2018). In the pea aphid, there is only one gene on the X chromosome that controls dispersal behavior, but this does not seem to be the case for most species (Pereira and Sokolowski 1993; Caillaud et al. 2002; Edelsparre et al. 2014). Butterflies show an oligogenic pattern (a small number of genes of large effect), in the form of an epistatic interaction between just 2 loci (Wheat et al. 2011; Niitepõld and Saastamoinen 2017). However, Jordan et al. (2012) showed that variation in locomotor behaviors in *Drosophila melanogaster* is associated with 192 genes, indicating a polygenic pattern.

*Tribolium castaneum* is an ideal system for understanding the genetic architecture of dispersal behavior. *Tribolium castaneum* is a cosmopolitan human commensal stored product pest. These beetles live in a variety of processed grains and frequently disperse to find new mates and resources. Previous studies have shown that dispersal behavior responds rapidly to selection and that males are more likely to disperse than females (Naylor 1961; Ritte and Lavie 1977; Lavie and Ritte 1978). Dispersal in *T. castaneum* has, also, been positively correlated with density dependence and fecundity (Naylor 1961; Ritte and Lavie 1977; Lavie and Ritte 1978). In contrast, resource availability is negatively correlated with dispersal tendency (Naylor 1961; Lavie and Ritte 1978). Dispersal tendency has even been shown to be impacted by interactions between species (higher emigration when the population size of other species is high) (Goodnight 1990a, 1990b, 2011). However, a full understating of how this behavior is inherited is lacking. We applied a line cross-analysis (LCA) approach to artificially selected lines to estimate the genetic architecture and inheritance of dispersal tendency.

The genes underlying traits can act in a variety of fashions. Additive effects are the simplest type of genetic effects and occur when the alleles at a locus contribute equally and independently of other loci in contributing to the observed phenotype. However, a variety of nonadditive effects (e.g., epistasis and maternal effects) are often found to underly complex traits. Epistatic effects describe a case where the contribution of an allele at one locus is dependent on the genotype at a second locus. Maternal effects are common and describe a case where an individual’s phenotype is influenced by the mother’s genotype or condition. The genetic architecture of traits can be investigated using a variety of methods. Genome-wide association studies (GWAS) and quantitative trait locus (QTL) analyses have become particularly popular in the last decade. GWAS and QTL studies are infamous for inferring an oligogenic genetic architecture for complex traits (Mackay et al. 2012; Husby et al. 2015; Santure et al. 2015). However, we know that these tests can lack the power to detect genes of small effect, complex architectures like epistasis, and parent of origin effects (Rockman 2012). However, we know that these complex architectures are common in determining many traits of biological interest including reproductive isolation, disease, and life history (Wagner et al. 1994; Leips and Mackay 2002; Moore 2003). Life-history traits, like dispersal, are expected to harbor more dominant and epistatic variance than additive variance making them more difficult to detect with GWAS and QTL studies alone (Roff and Emerson 2006: but see Houle 1992).

We chose to apply LCA which offers a fundamentally different approach to understanding the genetic underpinnings of traits. Rather than focusing on locus identification as GWAS and QTL do, LCA focuses on understanding the mode of gene action that is responsible for variation in the trait of interest. For instance, the genes responsible for variation in a trait could act in an additive fashion, through dominance, or epistasis, or even more complex modes of gene action like additive by sex interactions (Bulmer 1980; Lynch and Walsh 1997; Kearsey and Pooni 1998). Using artificial selection, we created lines for low and high dispersal tendency in *T. castaneum.* We found that dispersal tendency has both additive and epistatic components that are important in determining variation in the dispersal tendency.

## Methods

### Source Population

A population of *T. castaneum* was collected from a feed store in Hereford, Texas (34.80°, −102.40°) in 2014 (approximately 200 individuals). The beetles were housed in glass jars with 200 mL of standard media (95% whole wheat flour and 5% brewer’s yeast) in an incubator at 30°C with 60% humidity. Prior to the start of this study, these beetles were maintained by starting a new generation every 45 days by collecting approximately 200 adult beetles from an existing population jar and placing them in a new jar with fresh media. This 5-year period of acclimation to lab conditions ensures that the beetles were not impacted by adaptation to a lab environment during the present study.

### Measuring Dispersal Tendency

**Figure 1.**
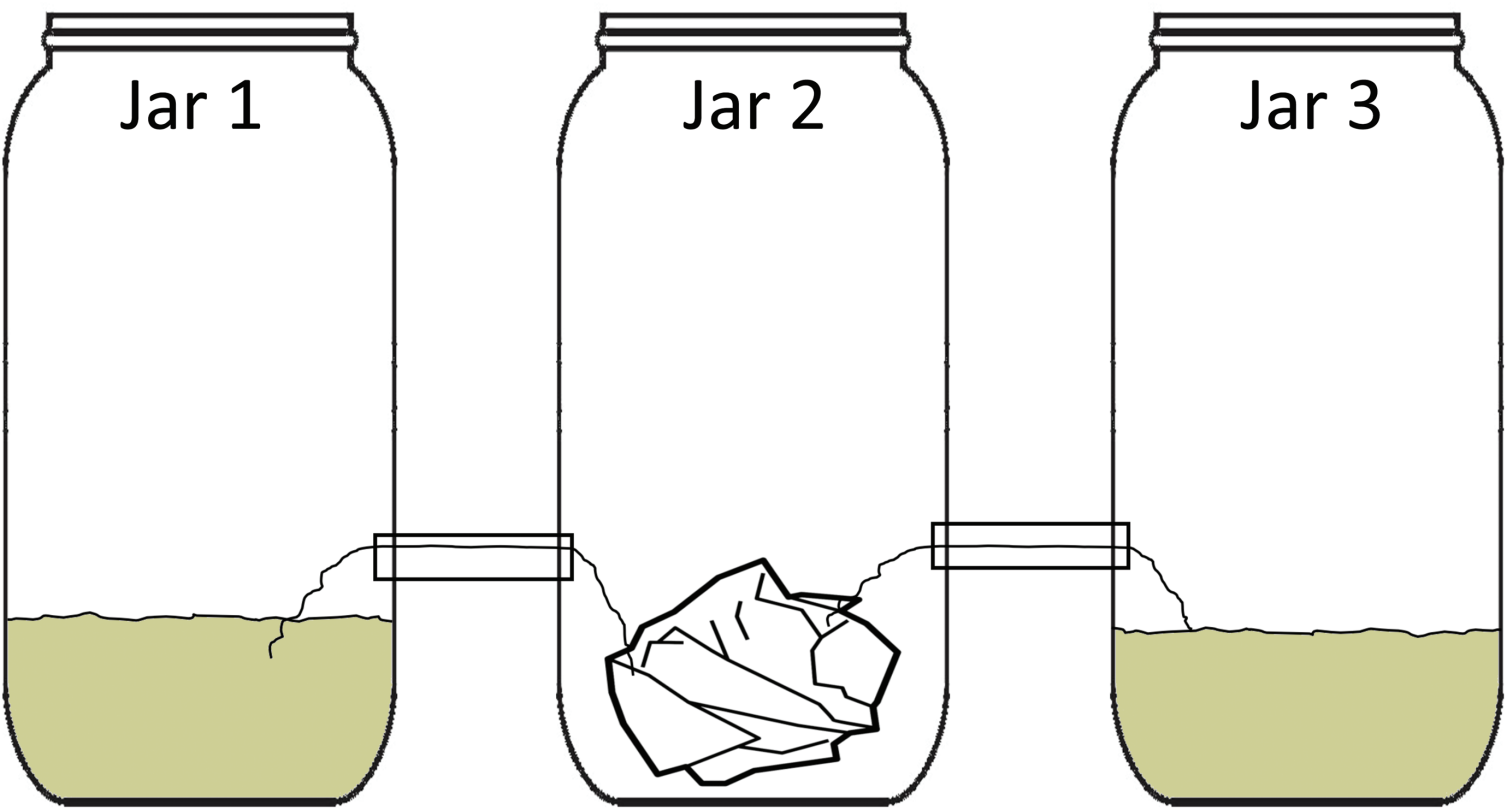
Experimental setup for dispersal selection. The beetles started in jar one containing the conditioned media. Beetles were classified as dispersers if they moved into jar 2 or jar 3 with the fresh media. Beetles were classified as non-dispersers if they remained in jar 1 during the experimental period.

To measure the dispersal tendency and perform artificial selection, we created a dispersal chamber inspired by previous work (Figure 1) (Arnold et al. 2017). This dispersal chamber consisted of 3 interconnected fly bottles that allow beetles the opportunity to disperse to connected jars and gain access to fresh media. Beetles were initially placed in the leftmost jar with 50 mL of conditioned media. This conditioned media consisted of flour that had been used for approximately 1 week by another population of beetles. To sterilize the media, it was baked for 2 hours at 125°C and then sifted to remove any detritus. The use of conditioned media has been shown to increase dispersal tendency, and this was chosen to ensure that a sufficient number of beetles would disperse, allowing for selection (Sokoloff 1977). To disperse, beetles climbed a 200 mm piece of string to the next jar through a 150 mm straw connecting the jars. The middle jar had a small tissue in the bottom to ensure that the beetles would not get stuck at the bottom and were able to move to the last jar. In the final jar, the beetles encountered 50 mL of fresh media. When we report dispersal tendency of a line, it is the proportion of the 200 beetles that had moved to jar 2 or jar 3 at the end of a 24-hour test period. We chose to count both jars 2 and 3 as dispersed beetles because the beetles could move between jars and had the opportunity to enter jar 3 and then return to jar 2. For all measurements of dispersal tendency, the dispersal chamber was kept undisturbed in an incubator ensuring that all lines had identical temperature, humidity, and light during phenotyping. All beetles had dispersal tendency measured within 1 week of eclosion (i.e., the transition from pupae to adult stage).

### Artificial Selection

Selection lines were initiated by collecting 1200 male and 1200 female pupae from the source population. These were subdivided into 3 replicates for the high selection lines and 3 replicates for the low selection lines (for each sex). Each of the 12 groups of 200 sexed pupae were kept isolated in 250 mL fly jars with standard media for up to 1 week to ensure that all of the pupae had become adults.

To perform selection, we placed 200 virgin beetles of the same sex in the conditioned media (Figure 1, jar 1) and allowed the beetles 24 hours to move between jars. The dispersal chamber was left undisturbed in the incubator during the 24-hour dispersal period. Parents for the low selection line were drawn from those beetles that remained in the conditioned media, while the high selection line was drawn from beetles that moved into either the second or third jars before the end of the 24-hour test. This was replicated to create 3 sets of high and low selection lines.

After the 24-hour test period, the selected males and females (approximately 80 to 100 each) from a replicate were combined and allowed to randomly mate. Parents were removed from the mating jar after 14 days and pupae for the next generation of selection were collected beginning on or about 25 days after the initiation of mating. This process was repeated for a total of 5 generations.

### Generation of Line Crosses

**Figure 2.**
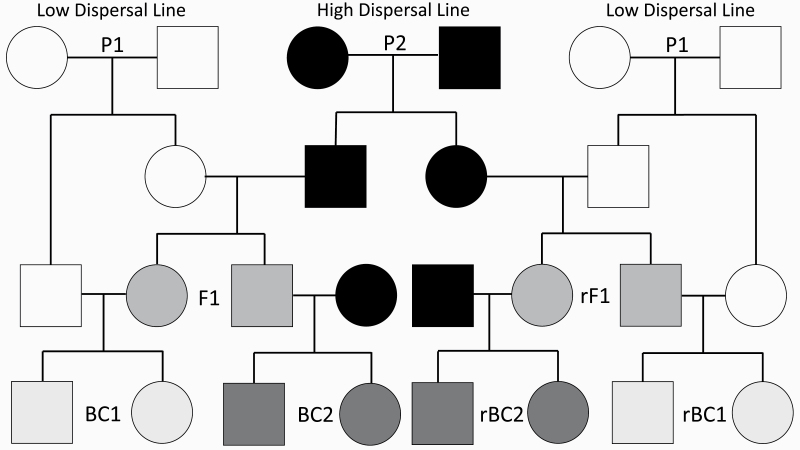
Crossing design used to generate study lines. The low dispersal line (P1) is shown on both the left and right of the plot to allow depiction of all crosses. The shade of each shape indicates the proportion of the genome that originates from each of these lines (pure P1 genome is colored in white while pure high dispersal line (P2) genome is colored black). In all cases, squares indicate sires while circles indicate dams. Each cross consisted of 3 replicates that are not depicted.

After 3 generations of selection 100 males and 100 females were drawn from each replicate selection line to act as our high dispersal line (P2) and low dispersal line (P1). When mating beetles for our line crosses we allowed adults to mate for 14 days after which adults were removed and larvae were allowed to develop. The offspring of each cross were sexed as pupae and kept isolated until all beetles had eclosed and then had dispersal tendency measured as described above. Parental lines (P1 and P2) were used to create both F1 and reciprocal F1 lines. F1s were then mated to both parental lines to generate a total of 8 lines. For each cross, both males and females were measured for dispersal tendency. All crosses are depicted in Figure 2. This set of crosses was replicated 3 times.

### Inference of Composite Genetic Effects

An LCA approach was used to infer the relative contribution of possible composite genetic effects (i.e., additive, dominance, epistatic, etc.) on divergence in dispersal tendency among our 2 selected lines. This method requires both a mean and standard error for each line included in the study. For each line, we used the 3 replicates to estimate the mean and standard error for a line.

Since each line will have different proportions of the parental genome and different levels of heterozygosity, the phenotype for each line has different opportunities to be impacted by possible composite genetic effects. For instance, the phenotype measured in the selected lines P1 and P2 should not be strongly impacted by dominance since they have undergone selection for alleles that contribute to either low or high dispersal tendency respectively. In contrast, the F1 line is expected to be strongly impacted by dominance since it is heterozygous at all loci that carry fixed differences in the parental lines. This characteristic allows us to construct a matrix of coefficients (C-matrix) that describes the opportunity for each possible genetic effect to impact dispersal tendency in each of our 8 lines (Lynch and Walsh 1997). Based on the crosses that we performed, we are able to construct a C-matrix with a total of 27 possible composite genetic effects. These genetic effects include 8 simple effects: additive effects from loci on autosomes, X, and Y chromosomes, dominance effects on autosomes and X chromosomes, cytotype, and maternal effects that are either additive or dominant. The remaining 19 composite genetic effects described in the C-matrix capture various possible pairwise epistatic interactions, for instance, autosomal additive by additive epistasis (the full C-matrix is given in Supplementary Table 1).

Using this information, we performed an LCA which is a weighted least squares model that allows us to represent a model of genetic architecture as a linear model (1)

$$\begin{array}{l} y=C\beta \;+e \end{array}$$ (1)

Where y represents the vector of observed line means, C is the C-matrix that describes the opportunity for each genetic effect to impact the phenotype of a line, β is the vector of parameters to be estimated that describe the degree to which each composite genetic effect is responsible for the observed line means, and e is a vector of the random errors associated with the means of each cohort. In the weighted least squares approach, we then find the estimate of the parameters $\hat{\beta }$ that minimizes the weighted sum of squares (2).

$$\begin{array}{l} {\left(y-C\beta \right)}^{T}V^{-1}(y-C\beta ) \end{array}$$ (2)

Here V is the variance-covariance matrix of e. In LCA, V is a diagonal matrix with the standard errors of cohort means along the diagonal. This scales each cohort’s contribution to the sum of squares by the certainty of the cohort mean. This means that if there is large uncertainty in a mean for a given line, it will contribute less to the sum of squares and by extension to the inference of the genetic architecture for the trait.

We used the software SAGA 2.0 to evaluate all possible models for the genetic architecture of our trait (Armstrong et al. 2019). Because we have a total of 8 lines, we limited the space of possible models to those with 7 or fewer parameters (composite genetic effects). This leads to a potential model space of 1.28 million models. A large proportion of these models will include genetic effects where the coefficients in the C-matrix are highly correlated leading to difficulty in calculating the maximum likelihood estimate of parameters. Models that exhibit this characteristic are dropped from the analysis and parameters are estimated by the remaining models. Previous simulation studies indicate that this does not lead to significant bias or loss of power in the inference of composite genetic effects under a model averaging approach (Blackmon and Demuth 2016; Armstrong et al. 2019). The AIC score for each evaluated model was recorded and we constructed a 95% confidence set of models that were used to produce model-averaged results that account for model selection uncertainty (Burnham and Anderson 2002).

To investigate the veracity of our LCA findings, we performed a forward time diploid, 20 loci, biallelic simulation. We assumed that dispersal tendency could be impacted by up to 20 unlinked loci (19 autosomal and 1 cytotype). To maximize the opportunity for transgressive segregation, we made all dispersal alleles dominant to non-dispersal alleles. Each locus could increase dispersal by 5% if it carried 1 or 2 copies of the dispersal allele. Under this model, a line fixed for all dispersal alleles would have 100% dispersal, and 1 fixed for all alternative alleles would have 0% dispersal.

To perform the simulation, we first generated a genome for the high dispersal line (P2) by randomly choosing 12 loci and fixing these sites for the dispersal alleles while the remaining 8 were fixed for the non-dispersal alleles. The low dispersal line (P1) genome was created similarly, but only 1 site was chosen at random and fixed for the dispersal alleles while all other sites were fixed for the non-dispersal alleles. Thus, our simulated P1 and P2 will have a dispersal tendency equal to what we observed in our empirical P1 and P2 lines.

Using these simulated genomes, we can draw gametes from the P1 and P2 lines (allowing for recombination between all loci and strict maternal inheritance of cytotype). These gametes were combined to generate 100 F1 and rF1 offspring. These F1 genomes were used to generate 100 offspring of each of the 4 backcrosses that we created in our experiment. We then calculated the mean and standard error for the dispersal phenotype for each of the 8 simulated lines. This data was, then, used to perform an LCA in the same fashion as described for the empirical data. This process was repeated 100 times to evaluate the impact of dispersion in an experiment like ours. All analyses and simulations were completed using R, version 3.6.3 (R Core Team 2020). Code and data to perform all analyses reported are available via GitHub https://github.com/coleoguy/dispersal.

## Results

### Response to Selection

Our selection lines showed an immediate but short-lived response to selection (Supplementary Figure S1). The base population had a mean dispersal tendency of 25%. Over 3 generations of selection, the dispersal in the P2 line increased to 59% while dispersal tendency in the P1 line reduced to 5%. However, continued selection led to little change; the means in the fifth generation were 70% and 18% for the P2 and P1 lines respectively. We have no comparable dispersal tendency measure for generation 4. In all other generations, lines were phenotyped approximately 7 days after the collection of pupae. However, in generation 4, phenotyping occurred 1 week later, and both P2 and P1 lines exhibited unusually high dispersal tendency. Further exploratory investigation revealed that dispersal behavior in *T. castaneum* appears to increase with time at least under our experimental conditions. One possible explanation is that we held beetles in single-sex populations and perhaps this leads to increased attempts to disperse when no mate has been located.

### Impact of Sex on Dispersal Tendency

In accordance with previous research, we found a consistent but weak effect of sex on dispersal tendency. To test the significance of this observation, we used the R package LME4 to fit a mixed model (Bates et al. 2015). Dispersal tendency was the response variable, sex was a fixed effect, and the line was a random effect; we used the standard link function and assumed normally distributed random effects. With this approach, we find that male dispersal tendency is approximately 5.0% higher than female dispersal tendency (*P*-value = 0.018, *T* = 2.48, df = 37; Supplementary Figure S2).

### Composite Genetic Effects

**Figure 3.**
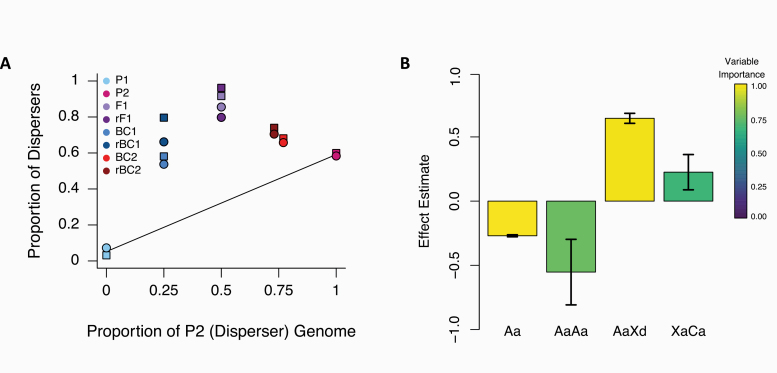
**(A)** Dispersal proportion among lines. On the horizontal axis, we show the proportion of the genome that originates from the P2 line. On the vertical axis, we show the proportion of dispersers in each line. Circles are used to indicate females and squares to indicate males. BC2 and rBC2 have been staggered slightly away from the true value of 0.75 on the horizontal axis to allow visualization of both crosses. **(B)** Composite genetic effects describing divergence in dispersal tendency among selection lines. The 4 composite effects identified are autosomal additive (Aa), autosomal additive by additive epistasis (AaAa), autosomal additive by X dominance epistasis (AaXd), and X additive by cytotype additive epistasis (XaCa). The color gradient indicates variable importance based on the 95% model confidence set. Only composite effects with variable importance greater than 0.15 and a mean with a confidence interval that does not include zero are shown.

Phenotyping of our experimental lines demonstrated an unexpected pattern of transgressive segregation where crosses between the P2 and P1 lines exhibited higher dispersal rates than either of the parental lines (Figure 3A). When evaluating all possible LCA models, we found that 71% exhibited high levels of correlation among predictor variables. This reduced the possible model space to be searched to just 369, 342 models. Despite the vast model space that might explain the genetic architecture of dispersal tendency in *T. castaneum*, we find that a group of just 10 models is necessary to construct a 95% confidence set (Supplementary Table 2). Based on model averaging across this set of 10 models, we find that only 4 composite genetic effects are inferred to have strong contributions to divergence in dispersal tendency. Autosomal additive, autosomal additive by X dominance epistasis, autosomal additive by additive epistasis, and X additive by cytotype epistasis each have confidence intervals that exclude zero and variable importance greater than 0.5 (Figure 3B). In this confidence set of models, a single model containing these 4 composite genetic effects provided 67% of the model probability. The remaining 9 models all exhibited much smaller model probabilities (each less than 0.05), and each of these 9 competing models contained autosomal additive and autosomal additive by X dominance epistasis as 2 of the 4 predictors. Examining all other possible genetic effects, we find that none have variable importance scores greater than 0.11 suggesting that they were only rarely included in models that fit the data well. Furthermore, all other variables had confidence intervals that overlapped zero indicating that they can explain little of the variation that we see in dispersal tendency and that the effect estimate was not consistent among models (Supplementary Figure S3).

To assess the adequacy of the 4 inferred composite genetic effects to explain our data, we examined the change in the Cox-Snell pseudo *R*^2^ for 4 models, beginning with the model that included only the composite genetic effect with the highest variable importance score, autosomal additive. Then we assessed the next 3 models, each time adding the composite genetic effect with the next highest variable importance score (autosomal additive by X dominance epistasis, autosomal additive by additive epistasis, and X chromosome additive by cytotype additive epistasis). These pseudo *R*^2^ values ranged from just 0.39 for the autosomal additive only model to 0.99 for the 4-parameter model given the highest weight in our confidence set. When we plotted the observed mean phenotypes and the expected mean phenotypes under this 4-parameter model (Supplementary Figures S4), we found a striking match between values. The rBC2 line displayed the largest residual difference between expected and observed values but even this was very slight (approximately 2%).

To evaluate the potential for dispersion to impact our results, we analyzed 100 simulated line cross datasets. Our simulation scenario was successful in producing line cross datasets that exhibited transgressive segregation. Of the 100 simulated datasets, 68 had F1 lines that had higher dispersal than the simulated P2 dispersal line. Across all simulated datasets, F1 lines had a dispersal tendency that was, on average, 2.5% higher than the simulated P2 disperser line. During LCA, 70% of datasets were inferred to have a significant additive component (variable importance score higher than 50% and an estimate that excluded zero). Within these 70 datasets, 30, also, were inferred to have a significant epistatic composite effect. However, these simulated epistatic effects were an order of magnitude smaller than inferred with the empirical data (Supplementary Figure S5). To compare the relative balance of epistatic and additive effects, we calculated an epistatic to additive ratio. This ratio was formed by summing the absolute value of all inferred epistatic effects and dividing by the absolute value of the inferred additive effect. For the empirical dataset, this value is 5.27. In contrast, for simulated datasets, the ratio ranged from zero to 0.33. This indicates that dispersion alone is unlikely to explain the large contribution of epistatic component that we infer from our empirical data.

## Discussion

The present study joins a host of others that concludes that dispersal is polygenic and has epistatic interactions (Wheat et al. 2011; Niitepõld and Saastamoinen 2017). Our findings reaffirm the hypothesis that many loci with small effects are contributing to complex traits like dispersal behavior (Rockman 2012; Saastamoinen et al. 2018). Our analysis also shows a consistent sex effect where males have higher dispersal tendency than females. This confirms previous work on *T. castaneum* dispersal (Naylor 1961; Ritte and Lavie 1977; Lavie and Ritte 1978). However, we note that our experimental design may have led to higher male dispersal than may be observed in more natural conditions. For instance, keeping beetles in single-sex virgin populations until phenotyping may lead to male beetles exhibiting higher dispersal tendency as they searched for mates.

One assumption of LCA is that there is no dispersion—all alleles that contribute to a higher phenotype are fixed in the high line, and all alleles contributing to a lower phenotype are fixed in the low line. In a short-term artificial selection experiment like ours, this assumption may not be satisfied. In our experiment, we observe transgressive segregation and epistatic composite genetic effects. Both of these characteristics can be due to the dispersion of alleles among the selection lines (Kearsey and Pooni 1998). To investigate whether dispersion could produce inferences similar to ours, we performed a forward time diploid, 20 loci, biallelic simulation.

Results from our simulation study showed that as expected dispersion can lead to false inferences of epistasis. Out of the 100 simulated line cross experiments, 31 supported a role for epistasis. However, the magnitude of epistasis that we infer in these simulated datasets is strikingly small compared to what we infer from our empirical data. Furthermore, the contribution of epistasis relative to additive variance is remarkably different in the empirical dataset relative to the simulated datasets. We suggest that these findings show that it is unlikely that the large role of epistasis that we infer is simply from dispersion among our parental lines. Furthermore, the relatively small role of epistasis that we infer from dispersion points to an added strength of accounting for model selection uncertainty during LCA not previously documented (Blackmon and Demuth 2016; Armstrong et al. 2019).

The rapid response to selection that we observed in our P1 and P2 lines suggests that there is an additive genetic variance that can be selected upon for dispersal tendency. However, the results of our LCA support a large role for epistatic interactions as the explanation for the total divergence in our 2 parental lines. Though these results may appear to conflict with one another initially, they are not at odds with previous analyses or theory concerning the expected standing genetic variation for traits, like dispersal, that may have a strong link to fitness (Mousseau and Roff 1987; Roff and Emerson 2006).

If a trait is directly linked to fitness, then, the additive genetic variance should erode relatively quickly as the fittest allele is fixed in the population (Curtsinger et al. 1994). In contrast, the epistatic variance may remain present in the population for long periods of time (Wade 1992). One avenue to investigate this is by comparing the genetic architecture of traits that vary in the degree to which they are connected to fitness. For instance, a morphological trait, like height, may have some correlation with fitness, but it is expected to be far less than the correlation between fecundity and fitness. A series of analyses comparing the genetic architecture of multiple morphological and life-history traits in a variety of species have been performed to investigate this (Roff and Mousseau 1987; Mousseau and Roff 1987; Houle 1992; McCleery et al. 2004; Roff and Emerson 2006). A clear pattern has emerged that morphological traits on average have a greater proportion of total genetic variance that is additive, while life-history traits have a greater proportion of total genetic variance that is epistatic (Mousseau and Roff 1987; Roff and Mousseau 1987).

Previous work in *T. castaneum* has shown a positive correlation between fitness and dispersal (Lavie and Ritte 1978). Therefore, we expect to see a greater proportion of dominance and epistatic variance due to past strong selection on this fitness-related trait, and our LCA findings are consistent with this expectation. However, if a trait has primarily epistatic variation, why do we also find a robust response to selection? We do not have a measure of additive variance in dispersal tendency for the original population; however, we suggest that during the sampling and selection process that the P1 and P2 lines experienced, the epistatic variance may have been converted to additive variance through randomly fixing one of the interacting loci allowing for a continued response to selection (Goodnight 1988). This would be consistent with both our response to selection and our inference of the composite genetic effects responsible for divergence in dispersal tendency among our selection lines.

Under many circumstances, the additive variation in a trait will determine the outcome of selection (Falconer and Mackay 1996). The alternative view that epistatic variation in traits is important in the course of evolution was a central theme in the work done by Wright (1940). Theoretical work has shown the importance of nonadditive effects on the evolution of a trait and that nonadditive effects can be converted to additive variance by a population bottleneck, which allows for further selection to act on the trait (Goodnight 1988; Keightley 1996; Fuerst et al. 1997; Łukaszewicz 2000; Soriano 2000; Wade 2002; Carlborg et al. 2006; Monnahan and Kelly 2015).

Our results show that even for a trait with little contribution from additive genetic effects, epistatic variance may be abundant and may contribute more to divergence in dispersal tendency. Because the conversion of epistatic variance to additive variance is made more likely when populations are small (Wade and Goodnight 1998), it seems reasonable that during the course of our selection experiment epistatic variance was converted to additive variance and fixed in the selection lines. A strong test of the importance of this process would be to replicate the experiment that we have performed but with multiple population sizes. Our expectation is that small populations should respond more rapidly as formerly epistatic variation is converted to additive variation and fixed. This would be strong evidence that epistatic variance may act as an adaptive reserve that is uncovered in small populations allowing a population to reach new adaptive peaks.

## Supplementary Material

esaa030_suppl_Supplementary_Material

## Data Availability

Code and data to perform all analyses reported are available via GitHub https://github.com/coleoguy/dispersal.

## References

[CIT0001] Armstrong A, Anderson NW, Blackmon H. 2019. Inferring the potentially complex genetic architectures of adaptation, sexual dimorphism and genotype by environment interactions by partitioning of mean phenotypes. J Evol Biol. 32:369–379. 30698300 10.1111/jeb.13421

[CIT0002] Arnold PA, Rafter MA, Malekpour R, Cassey P, Walter GH, White CR. 2017. Investigating movement in the laboratory: dispersal apparatus designs and the red flour beetle, *Tribolium castaneum*. Entomol Exp Appl. 163:93–100.

[CIT0003] Bates D, Mächler M, Bolker B, Walker S. 2015. Fitting linear mixed-effects models using lme4. J Stat Softw. 67: 1– 48.

[CIT0004] Blackmon H, Demuth JP. 2016. An information-theoretic approach to estimating the composite genetic effects contributing to variation among generation means: Moving beyond the joint-scaling test for line cross analysis: I-T Approach to Line Cross Analysis. Evolution 70:420–432. 26704183 10.1111/evo.12844

[CIT0005] Bulmer MG. 1980. The mathematical theory of quantitative genetics. Oxford (UK): Clarendon Press.

[CIT0006] Burnham KP, Anderson DR. 2002. Model selection and multimodel inference. New York: Springer.

[CIT0007] Caillaud MC, Boutin M, Braendle C, Simon J-C. 2002. A sex-linked locus controls wing polymorphism in males of the pea aphid, *Acyrthosiphon pisum* (Harris). Heredity 89:346–352. 12399992 10.1038/sj.hdy.6800146

[CIT0008] Carlborg Ö, Jacobsson L, Åhgren P, Siegel P, Andersson L. 2006. Epistasis and the release of genetic variation during long-term selection. Nat. Genet. 38:418–420. 16532011 10.1038/ng1761

[CIT0009] Clobert J, Baguette M, Benton TG, Bullock JM. 2012. Dispersal ecology and evolution. Oxford (UK): Oxford University Press.

[CIT0010] Curtsinger JW, Service PM, Prout T. 1994. Antagonistic pleiotropy, reversal of dominance, and genetic polymorphism. Am. Nat. 144:210–228.

[CIT0011] Drangsholt TMK, Damsgård B, Olesen I. 2014. Quantitative genetics of behavioral responsiveness in Atlantic cod (*Gadus morhua* L.). Aquaculture 420–421:282–287.

[CIT0012] Edelsparre AH, Vesterberg A, Lim JH, Anwari M, Fitzpatrick MJ. 2014. Alleles underlying larval foraging behaviour influence adult dispersal in nature. Ecol Lett. 17:333–339. 24386971 10.1111/ele.12234

[CIT0013] Falconer DS, Mackay TFC. 1996. Introduction to quantitative genetics. Essex (UK): Longmans Green, Harlow.

[CIT0014] Fuerst C, James JW, Sölkner J, Essl A. 1997. Impact of dominance and epistasis on the genetic make-up of simulated populations under selection: a model development. J Anim Breed Genet. 114:163–175. 21395812 10.1111/j.1439-0388.1997.tb00502.x

[CIT0015] Goodnight CJ. 1988. Epistasis and the effect of founder events on the additive genetic variance. Evolution. 42:441–454. 28564006 10.1111/j.1558-5646.1988.tb04151.x

[CIT0016] Goodnight CJ. 1990a. Experimental studies of community evolution I: the response to selection at the community level. Evolution. 44:1614–1624. 28564309 10.1111/j.1558-5646.1990.tb03850.x

[CIT0017] Goodnight CJ. 1990b. Experimental studies of community evolution II: the ecological basis of the response to community selection. Evolution. 44:1625–1636. 28564304 10.1111/j.1558-5646.1990.tb03851.x

[CIT0018] Goodnight CJ. 2011. Evolution in metacommunities. Philos Trans R Soc Lond B Biol Sci. 366:1401–1409. 21444314 10.1098/rstb.2010.0290 PMC3081572

[CIT0019] Houle D. 1992. Comparing evolvability and variability of quantitative traits. Genetics. 130:195–204. 1732160 10.1093/genetics/130.1.195 PMC1204793

[CIT0020] Husby A, Kawakami T, Rönnegård L, Smeds L, Ellegren H, Qvarnström A. 2015. Genome-wide association mapping in a wild avian population identifies a link between genetic and phenotypic variation in a life-history trait. Proc Biol Sci. 282:20150156. 25833857 10.1098/rspb.2015.0156 PMC4426624

[CIT0021] Jordan KW, Craver KL, Magwire MM, Cubilla CE, Mackay TF, Anholt RR. 2012. Genome-wide association for sensitivity to chronic oxidative stress in Drosophila melanogaster. PLoS One. 7:e38722. 22715409 10.1371/journal.pone.0038722 PMC3371005

[CIT0022] Kearsey MJ, Pooni HS. 1998. The genetical analysis of quantitative traits. Cheltenham (UK): Stanley Thornes (Publishers) Ltd.

[CIT0023] Keightley PD. 1996. Metabolic models of selection response. J Theor Biol. 182:311–316. 8944163 10.1006/jtbi.1996.0169

[CIT0024] Lavie B, Ritte U. 1978. The relation between dispersal behavior and reproductive fitness in the flour beetle *Tribolium castaneum*. Can J Genet Cytol. 20:589–595.

[CIT0025] Leips J, Mackay TF. 2002. The complex genetic architecture of Drosophila life span. Exp Aging Res. 28:361–390. 12227919 10.1080/03610730290080399

[CIT0026] Łukaszewicz M. 2000. Non-additive genetic effects in animal selection. J Appl Genet. 42, 467–478. 14564022

[CIT0027] Lynch M, Walsh B. 1997. Genetics and analysis of quantitative traits. Sunderland (MA): Sinauer Associates Incorporated.

[CIT0028] Mackay TFC, Richards S, Stone EA, Barbadilla A, Ayroles JF, Zhu D, Casillas S, Han Y, Magwire MM, Cridland JM, et al. 2012. The *Drosophila melanogaster* genetic reference panel. Nature 482:173–178. 22318601 10.1038/nature10811 PMC3683990

[CIT0029] McCleery RH, Pettifor RA, Armbruster P, Meyer K, Sheldon BC, Perrins CM. 2004. Components of variance underlying fitness in a natural population of the great tit Parus major. Am Nat. 164:E62–E72. 15478083 10.1086/422660

[CIT0030] Monnahan PJ, Kelly JK. 2015. Epistasis is a major determinant of the additive genetic variance in *Mimulus guttatus*. PLoS Genet. 11:e1005201. 25946702 10.1371/journal.pgen.1005201 PMC4422649

[CIT0031] Moore JH. 2003. The ubiquitous nature of epistasis in determining susceptibility to common human diseases. Hum Hered. 56:73–82. 14614241 10.1159/000073735

[CIT0032] Mousseau TA, Roff DA. 1987. Natural selection and the heritability of fitness components. Heredity (Edinb). 59 (Pt 2):181–197. 3316130 10.1038/hdy.1987.113

[CIT0033] Naylor AF. 1961. Dispersal in the red flour beetle *Tribolium castaneum* (Tenebrionidae). Ecology. 42:231–237.

[CIT0034] Niitepõld K, Saastamoinen M. 2017. A candidate gene in an ecological model species: Phosphoglucose Isomerase (Pgi) in the Glanville Fritillary Butterfly *(Melitaea cinxia)*. Ann. Zool. Fenn. 54:259–273.

[CIT0035] Pereira HS, Sokolowski MB. 1993. Mutations in the larval foraging gene affect adult locomotory behavior after feeding in *Drosophila melanogaster*. Proc Natl Acad Sci U S A. 90:5044–5046. 8506349 10.1073/pnas.90.11.5044 PMC46650

[CIT0036] R Core Team, 2020. R: A Language and Environment for Statistical Computing. Vienna (Austria): R Foundation for Statistical Computing.

[CIT0037] Ritte U, Lavie B. 1977. The genetic basis of dispersal behavior in the flour beetle *Tribolium castaneum*. Can. J. Genet. Cytol. 19:717–722.

[CIT0038] Rockman MV. 2012. The QTN program and the alleles that matter for evolution: all that’s gold does not glitter. Evolution. 66:1–17. 22220860 10.1111/j.1558-5646.2011.01486.x PMC3386609

[CIT0039] Roff DA, Emerson K. 2006. Epistasis and dominance: evidence for differential effects in life-history versus morphological traits. Evolution. 60:1981–1990. 17133855

[CIT0040] Roff DA, Mousseau TA. 1987. Quantitative genetics and fitness: lessons from Drosophila. Heredity (Edinb). 58 (Pt 1):103–118. 3818341 10.1038/hdy.1987.15

[CIT0041] Ronce O. 2007. How does it feel to be like a rolling stone? Ten questions about dispersal evolution. Annu. Rev. Ecol. Evol. Syst. 38:231–253.

[CIT0042] Saastamoinen M, Bocedi G, Cote J, Legrand D, Guillaume F, Wheat CW, Fronhofer EA, Garcia C, Henry R, Husby A, et al. 2018. Genetics of dispersal. Biol Rev Camb Philos Soc. 93:574–599. 28776950 10.1111/brv.12356 PMC5811798

[CIT0043] Santure AW, Poissant J, De Cauwer I, van Oers K, Robinson MR, Quinn JL, Groenen MA, Visser ME, Sheldon BC, Slate J. 2015. Replicated analysis of the genetic architecture of quantitative traits in two wild great tit populations. Mol Ecol. 24:6148–6162. 26661500 10.1111/mec.13452 PMC4738425

[CIT0044] Sokoloff A. 1977. The biology of *Tribolium* with special emphasis on genetic aspects. Oxford (UK): Clarendon Press.

[CIT0045] Soriano JMV. 2000. Components of variation of polygenic systems with digenic epistasis. Genet Mol Biol. 23:883–892.

[CIT0046] Travis JMJ, Delgado M, Bocedi G, Baguette M, Bartoń K, Bonte D, Boulangeat I, Hodgson JA, Kubisch A, Penteriani V, et al. 2013. Dispersal and species’ responses to climate change. Oikos 122:1532–1540.

[CIT0047] Wade MJ. 1992. Sewall Wright: gene interaction and the shifting balance theory. In: Oxford surveys in evolutionary biology: volume 8: 1991. Oxford (UK): Oxford University Press. p. 32–65.

[CIT0048] Wade MJ. 2002. A gene’s eye view of epistasis, selection and speciation. J Evol Biol. 15:337–346.

[CIT0049] Wade MJ, Goodnight CJ. 1998. Perspective: the theories of fisher and wright in the context of metapopulations: when nature does many small experiments. Evolution. 52:1537–1553. 28565332 10.1111/j.1558-5646.1998.tb02235.x

[CIT0050] Wagner A, Wagner GP, Similion P. 1994. Epistasis can facilitate the evolution of reproductive isolation by peak shifts: a two-locus two-allele model. Genetics. 138:533–545. 7828834 10.1093/genetics/138.2.533 PMC1206169

[CIT0051] Wheat CW, Fescemyer HW, Kvist J, Tas E, Vera JC, Frilander MJ, Hanski I, Marden JH. 2011. Functional genomics of life history variation in a butterfly metapopulation. Mol Ecol. 20:1813–1828. 21410806 10.1111/j.1365-294X.2011.05062.x

[CIT0052] Wright S. 1940. Breeding structure of populations in relation to speciation. Am Nat. 74:232–248.

