# Supplementary material for: The March of the Beetles: Epistatic Components Dominate Divergence in Dispersal Tendency in Tribolium castaneum
Source: J Hered. 2020 Aug 14;111(5):498–505. doi: 10.1093/jhered/esaa030 (PMC7525825; doi:10.1093/jhered/esaa030)
Supplement: esaa030_suppl_Supplementary_Material [file esaa030_suppl_supplementary_material.pdf]

Supplement

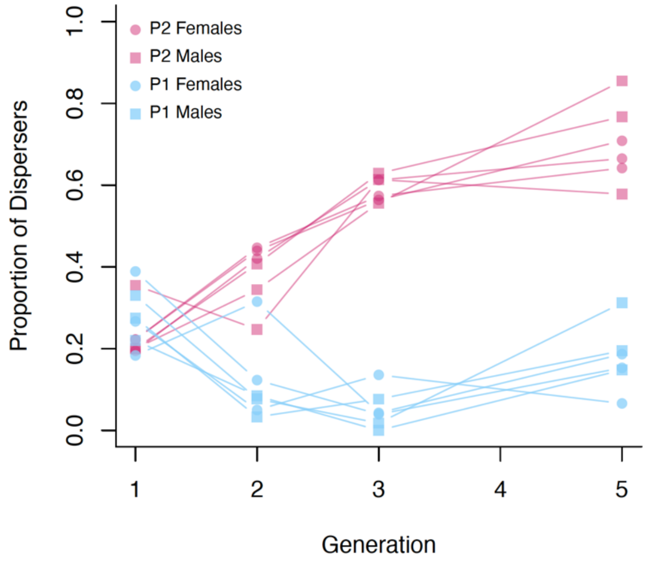

Figure S1. Response to selection on dispersal. The plot shows the response to selection in males (squares) and females (circles). The P2 high dispersal line is indicated in pink while the P1 low dispersal line is shown in blue. The plot shows that the response to selection has decreased between the first and second generations indicating that dispersal and non-dispersal alleles had fixed in the lines. The third generation was omitted from the graph because that generation was tested a week later than all other generations.

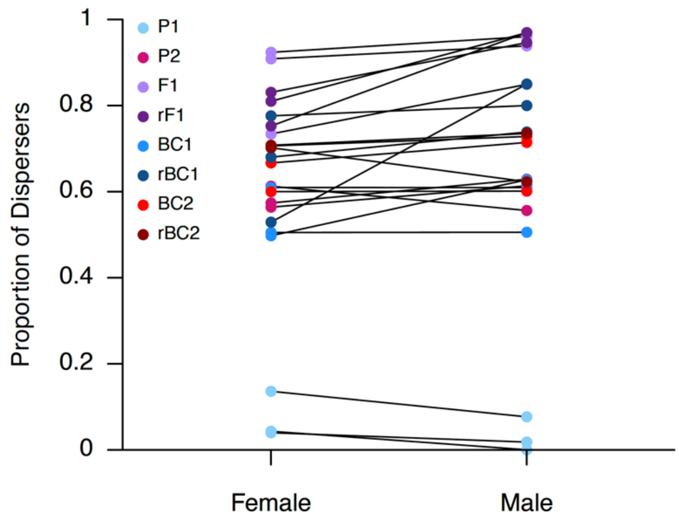

Figure S2. Dispersal tendency based on sex. The horizontal axis represents the sex of the beetles, while the vertical axis represents the mean proportion of dispersers for each line. Since most of the lines connecting females and males have a positive slope, we can see the sex effect. Males disperse more than females.

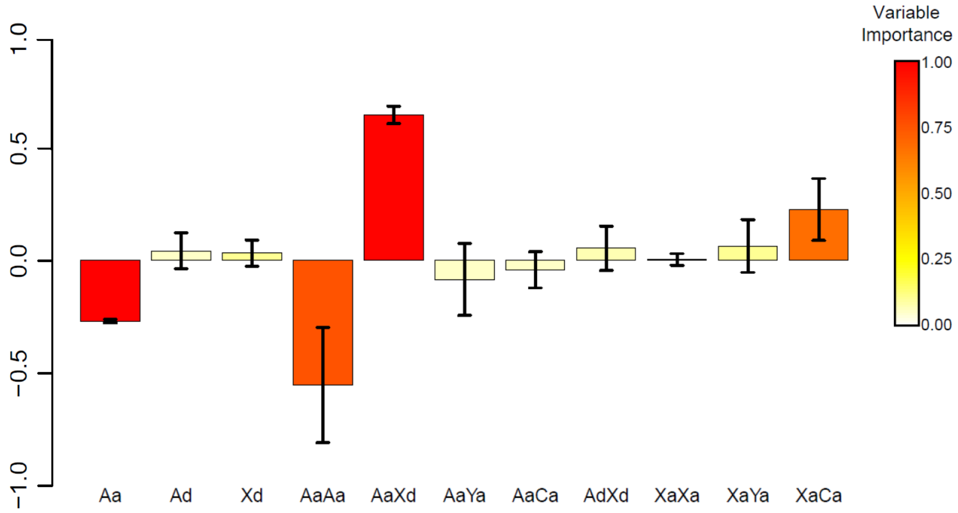

Figure S3. Composite genetic effects describing divergence in dispersal proportion among selection lines. The color gradient indicates variable importance based on the 95% model confidence set. Only composite effects with variable importance greater than 0.05 are shown.

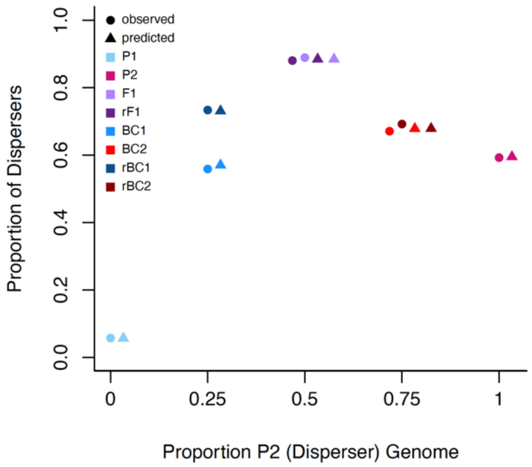

Figure S4. Expectation plot. On the horizontal axis, we plot the proportion of each lines genome that originates with the high dispersal line and on the vertical axis we plot the mean proportion of dispersers in each line. Circles indicate observed values while triangles indicated values predicted under the model with the highest Akaike weight. Points have been jittered in the horizontal to allow for visualization of all points.

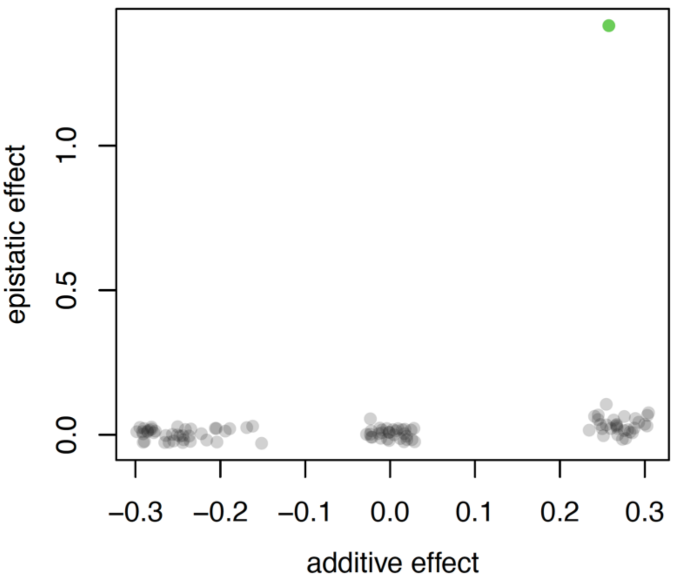

Figure S5 Comparison of simulated and empirical results. The vertical axis shows the sum of the absolute value of all significant epistatic effects, while the horizontal axis shows the significant additive effects. Each gray point represents results from a simulated dataset while the lone green point in the upper right quadrant represents results from the empirical data.

	P1	P2	F1	rF1	BC1	BC2	rBC1	rBC2
Aa	1	-1	0	0	0.5	-0.5	0.5	-0.5
Ad	0	0	1	1	0.5	0.5	0.5	0.5
Xa	1	-1	0.5	-0.5	0.25	-0.5	0.5	-0.25
Xd	0	0	0.5	0.5	0.25	0.5	0.5	0.25
Ya	0.5	-0.5	-0.5	0.5	0.5	-0.5	0.5	-0.5
Ca	1	-1	1	-1	1	-1	1	-1
Mea	1	-1	1	-1	0	-1	1	0
Med	0	0	0	0	1	0	0	1
AaAa	1	1	0	0	0.25	0.25	0.25	0.25
AaAd	0	0	0	0	0.25	-0.25	0.25	-0.25
AaXa	1	1	0	0	0.125	0.25	0.25	0.125
AaXd	0	0	0	0	0.125	-0.25	0.25	-0.125
AaYa	0.5	0.5	0	0	0.25	0.25	0.25	0.25
AaCa	1	1	0	0	0.5	0.5	0.5	0.5
AdAd	0	0	1	1	0.25	0.25	0.25	0.25
AdXa	0	0	0.5	-0.5	0.125	-0.25	0.25	-0.125
AdXd	0	0	0.5	0.5	0.125	0.25	0.25	0.125
AdYa	0	0	-0.5	0.5	0.25	-0.25	0.25	-0.25
AdCa	0	0	1	-1	0.5	-0.5	0.5	-0.5
XaXa	1	1	0.25	0.25	0.0625	0.25	0.25	0.0625
XaXd	0	0	0.25	-0.25	0.0625	-0.25	0.25	-0.0625
XaYa	0.5	0.5	-0.25	-0.25	0.125	0.25	0.25	0.125
XaCa	1	1	0.5	0.5	0.25	0.5	0.5	0.25
XdXd	0	0	0.25	0.25	0.0625	0.25	0.25	0.0625
XdYa	0	0	-0.25	0.25	0.125	-0.25	0.25	-0.125
XdCa	0	0	0.5	-0.5	0.25	-0.5	0.5	-0.25
YaCa	0.5	0.5	-0.5	-0.5	0.5	0.5	0.5	0.5

Supplemental Table 1. Matrix coefficients of composite genetic effects. The genetic effects are abbreviated with an initial letter indicating the genomic region of the effect: “A”, “X”, “Y”, or “M” for autosomal, X chromosome, Y chromosome or maternal effect respectively. This is followed by a lower case “a” or “d” indicating additive or dominance. For example, Aa indicates an autosomal additive. These abbreviations are combined to describe epistatic terms. For example, AdXa indicates autosomal dominance by X chromosome additive epistasis.

Model Weight	AIC	Aa (0.997)	AaXd (0.986)	AaAa (0.763)	XaCa (0.683)	XaYa (0.109)	Xd (0.109)	AdXd (0.084)	XaXa (0.082)	Ad (0.055)	AaYa (0.055)	AaCa (0.055)
0.668	-46.500	X	X	X	X							
0.041	-40.909	X	X					X	X			
0.041	-40.909	X	X	X				X				
0.041	-40.909	X	X	X					X			
0.027	-40.083	X	X			X				X		
0.027	-40.083	X	X			X					X	
0.027	-40.083	X	X			X						X
0.027	-40.083	X	X				X			X		
0.027	-40.083	X	X				X				X	
0.027	-40.083	X	X				X					X
Effect estimate		-0.27	0.645	-0.553	0.225	0.063	0.032	0.053	0.003	0.042	-0.084	-0.042

Supplemental Table 2. Confidence model set. Each row in the table indicates a model. The parameters included in the model are indicated by “X” in a column of the table. Parameter names are followed by their variable importance scores in parentheses. The effect estimates are based on the model averaging result. Effect estimates that are black had variable confidence intervals that exclude zero while those in grey did not.
